# ‘HeART of Stroke (HoS)’, a community-based Arts for Health group intervention to support self-confidence and psychological well-being following a stroke: protocol for a randomised controlled feasibility study

**DOI:** 10.1136/bmjopen-2015-008888

**Published:** 2015-08-04

**Authors:** Caroline Ellis-Hill, Fergus Gracey, Sarah Thomas, Catherine Lamont-Robinson, Peter W Thomas, Elsa M R Marques, Mary Grant, Samantha Nunn, Robin P I Cant, Kathleen T Galvin, Frances Reynolds, Damian F Jenkinson

**Affiliations:** 1 Faculty of Health and Social Sciences, Bournemouth University, Bournemouth, Dorset, UK; 2 Department of Clinical Psychology, University of East Anglia, Norwich, UK; 3 Oliver Zangwill Centre, Cambridgeshire Community Services NHS Trust, Ely, Cambridgeshire, UK; 4 School of Social and Community Medicine, University of Bristol, Bristol, UK; 5 Stroke Research, Royal Bournemouth and Christchurch Hospitals NHS Foundation Trust, Bournemouth, Dorset, UK; 6 Service User (formerly of Canterbury Christ Church University), Kent, UK; 7 Faculty of Health and Social Care, University of Hull, Hull, Yorkshire, UK; 8 College of Health and Life Sciences, Brunel University London, UK; 9 Stroke Unit, Royal Bournemouth and Christchurch Hospitals NHS Foundation Trust, Bournemouth, Dorset, UK

**Keywords:** STROKE MEDICINE

## Abstract

**Introduction:**

Over 152 000 people in the UK have strokes annually and a third experience residual disability. Low mood also affects a third of stroke survivors; yet psychological support is poor. While Arts for Health interventions have been shown to improve well-being in people with mild-to-moderate depression post-stroke, their role in helping people regain sense of self, well-being and confidence has yet to be evaluated. The main aim of this study is to explore the feasibility of conducting a pragmatic multicentre randomised controlled trial to assess the effectiveness and cost-effectiveness of an Arts for Health group intervention (‘HeART of Stroke’ (HoS)) for stroke survivors. HoS is a 10-session artist-facilitated group intervention held in the community over 14 weeks. It offers a non-judgemental, supportive environment for people to explore sense of self, potentially enhancing well-being and confidence.

**Methods and analysis:**

Sixty-four people, up to 2 years post-stroke, recruited via secondary care research staff or community stroke/rehabilitation teams in two UK centres will be randomised to either HoS plus usual care or usual care only. Self-reported outcomes, measured at baseline and approximately 5 months postrandomisation, will include stroke-related, well-being, mood, self-esteem, quality of life and process measures. Analyses will focus on estimating key feasibility parameters (eg, rates of recruitment, retention, intervention attendance). We will develop outcome and resource use data collection methods to inform an effectiveness and cost-effectiveness analysis in the future trial. Interviews, with a sample of participants, will explore the acceptability of the intervention and study processes, as well as experiences of the HoS group.

**Ethics and dissemination:**

National Health Service (NHS), Research and Development and University ethical approvals have been obtained. Two peer-reviewed journal publications are planned plus one service user led publication. Findings will be disseminated at key national conferences, local stakeholder events and via institutional websites.

**Trial registration number:**

ISRCTN99728983.

**Strengths and limitations of this study**

- Considers psychological aspects currently not addressed following stroke.
- Tests two different participant identification and recruitment routes (via stroke ward research staff in one centre, and community stroke and rehabilitation teams in the second centre).
- Explores the feasibility of using standardised outcome measures with participants who might have communication difficulties.
- Incorporates mixed methods, a feasibility economic component and assessor blinding
- Only includes a short-term follow-up (1 month post-HeART of Stroke intervention).
- Will inform the design and conduct of a fully powered randomised controlled trial (RCT).

## Introduction

Over 152 000 people experience a stroke in the UK each year^1^ and one-third are left with residual disabilities, including paralysis of one side, communication difficulties and cognitive difficulties.^2^ Psychological support post-stroke is still in its infancy. In a review of progress in improving stroke care, psychological support was rated as the least satisfactory service in long-term care.^3^ In the context of the Stroke Improvement Programme, Kneebone and Lincoln concluded that much needs to be done to both prevent and treat psychological problems after stroke.^4^

Low mood and depression are common and serious consequences of stroke, affecting approximately one-third of all stroke survivors,^5^ with peak incidence occurring after discharge to the community^6^ leading to potential adverse consequences, including increased mortality^7^ and poor participation in/reduced gains from rehabilitation.^8^ ^9^ If untreated, mood disturbance is associated with higher rates of mortality, long-term disability, hospital readmissions, suicide and higher utilisation of outpatient services.^10^ This can create long-terms costs not only for the stroke survivor, but also for family members^11^ ^12^ and increased costs for government, health and social services through reduced family employment and increased social and primary care needs.^13^

Medication has been shown to have a small but significant effect on depressive symptoms for people with stroke; however, adverse events can be high.^14^ A Cochrane review of psychotherapy for depressive symptoms after stroke found no evidence of benefit^15^ and cognitive behaviour therapy^16^ ^17^ has limited support; however, initial findings from motivational interviewing studies look promising^18^ and a trial is currently being carried out to explore an intervention involving motivational interviewing, grief resolution and psychoeducation.^19^ Counselling and psychotherapy are usually provided on an individual basis, but are reliant on good verbal communication which can limit their use—as one-third of people have a communication disability following stroke.^2^ ^20^

Three systematic reviews of the experiences of people following a stroke^21–23^ and a recent thematic synthesis^24^ have highlighted that people face existential challenges in terms of uncertainty and loss of their usual everyday world. People report a need to ‘get their lives back’. Failure to do so is associated with loss of confidence^25^; people having difficulty in ‘feeling part of things’^26^; becoming socially isolated^27^ and experiencing mood disturbance, as highlighted above.^28^

Separate theoretical models, based on empirical evidence, have been developed by Ellis-Hill *et al*,^29^ and Gracey *et al*^30^ to understand the processes involved in re-establishing a positive sense of self and confidence in life following a stroke. Key is being able to reconstruct a sense of meaning, predictability and coherence in everyday life when previously taken for granted assumptions no longer hold true, and developing new ways of ‘being in the world’.^31^ ^32^ Through the use of the imagination,^33^ Arts for Health (AfH) practices offer the opportunity for self-development using internal resources not usually available in everyday life.^34^ ^35^ Within a group setting, a collective sense of identification and belonging facilitates the process of self-development and acceptance.^36^ ^37^ This instils a sense of self-confidence, despite facing unfamiliarity, allowing people to get their lives ‘back on track’ and maintain or develop a sense of well-being.^38^

The James Lind Alliance top 10 priorities chosen by people with stroke, carers and health professionals includes ‘*Finding the best ways to improve confidence after stroke’.*^39^ The commitment to assign equal weight to both mental and physical health, and support well-being through the life course is key government policy (*Healthy Lives, Healthy People*^40^; *No Health without Mental Health*^41^). Specifically, the *National Stroke Strategy*^2^ and *Psychological Care after Stroke*^10^ published by the National Health Service (NHS) Stroke Improvement Programme have highlighted that psychological health and well-being need to be deemed nationally important outcomes in stroke rehabilitation. Interventions are required that can be accessed by all people (including those with communication difficulties) and that can be provided in cost-effective group settings.

While there are Cochrane reviews in areas related to Arts for Health (the use of music and the use of dance in depression^42^ ^43^) and research exploring the use of arts for stroke survivors in hospital settings,^44^ ^45^ there is a lack of research undertaken in the community.^46^ Using Arts for Health practices to support well-being is becoming increasingly recognised within the NHS with the Arts for Health movement^47^ and in policy with the creation of the All Party Parliamentary Group for Arts, Health and Wellbeing. Practices have been embedded nationally in the NHS in Arts on Prescription schemes.^48^ ^49^ The ‘Art Lift’ intervention in general practitioner (GP) practices has been shown to increase well-being^50^ and research suggests Arts on Prescriptions schemes lead to enhanced self-confidence and motivation.^51^ AfH approaches can reduce stigma and social isolation, and enhance self-esteem, confidence and well-being.^52–55^ In the current research, an Arts for Health approach^47^ will be used to create a group atmosphere where people feel safe to explore ‘hidden’ psychological aspects (low self-esteem, loss of confidence) so that they can tap into their personal resources to help them ‘get on’ with their lives.

If successful, Arts for Health groups have the potential to lead to improved mood and confidence, providing longer term NHS savings in terms of increased participation, better response to rehabilitation, reduced hospital readmissions and reduced utilisation of outpatient/primary care services. A review of the use of the arts following stroke reported positive outcomes from case studies and called for well-conducted mixed methods research.^46^ A recent report by the Arts Council England^56^ highlights the health and well-being benefits of the arts and the need for larger sample sizes, longitudinal studies and experimental methods. Hackett *et al*^57^ call for trials of the delivery of psychological stroke interventions by trained health professionals or lay people following hospital discharge.

The longer term aim of this research programme is to test the effectiveness and cost-effectiveness of an arts for health intervention in the community.

## Methods and analysis

### Aim

The aim of this feasibility study is to inform the planning of a definitive multicentre randomised controlled trial. In the current study we will explore key trial processes, and the feasibility and acceptability of a community Arts for Health group intervention (‘HeART of Stroke’ group). The aim of the future trial will be to test the effectiveness and cost effectiveness of the HeART of Stroke group intervention, provided in addition to usual care, to support self-confidence and psychological well-being following stroke compared with usual care alone.

### Objectives

The objectives of this feasibility study are to:

1. Assess the acceptability of key aspects of study design, randomisation and recruitment processes, and of the HoS group intervention by participants, healthcare staff and facilitators;
2. Estimate recruitment and short-term retention rates;
3. Estimate HoS group attendance rates;
4. Assess the suitability of the outcome measures and feasibility of the assessment strategy;
5. Refine the selection of the outcome measures; in particular, to help inform the selection of the primary outcome for the full scale RCT;
6. Explore qualitatively individuals’ experiences of participating in the study and gather feedback about the intervention and outcome measures;
7. Collect data on the variability of outcome measures to inform a sample size calculation for a larger trial and obtain a preliminary estimate of effect size;
8. Refine the HoS group intervention and its delivery;
9. Explore differences in processes between the two study centres;
10. Identify, measure and value resources required to deliver the intervention in the community;
11. Develop data collection tools to measure resource use in the follow-up period to inform the design of a future within-trial economic evaluation with minimal missing data.

### Study design

**Figure 1:**
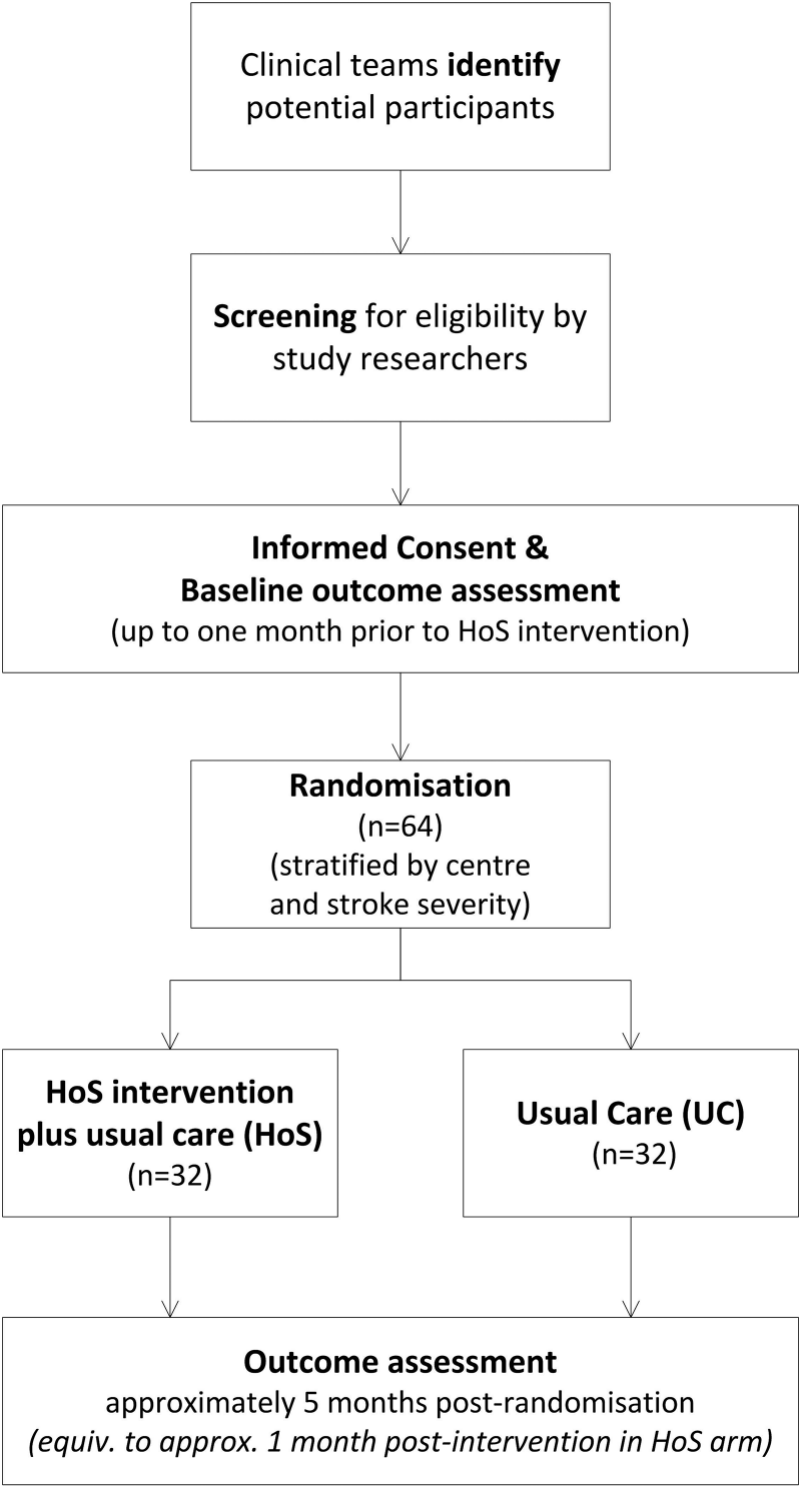
HeART of Stroke (HoS) study flow chart.

This is a 24-month two-centre parallel arm pragmatic randomised controlled feasibility study of a HoS group plus usual care compared with usual care alone (see figure 1), with nested economic and qualitative components. We are using a usual care comparator in order that the future trial will reflect real-world effectiveness of this complex intervention. A nested qualitative interview study will provide insights into individuals’ experiences of participating and the acceptability of study processes, the HoS intervention and outcome measures. The feasibility economic component will enable us to pilot methods to collect intervention costs and resource utilisation to inform the future trial design and within-trial economic evaluation. These trial processes will be tested for a follow-up period of 5 months postrandomisation. The definitive trial would include longer follow-up to capture the longer term health and economic benefits of treatment delivery.

### Service user involvement

Public and service users were involved in the design of the study, the development of the funding application, the design of the HoS intervention, the selection of relevant outcome measures and the design of data collection tools. This work was instigated by RC, a stroke survivor and co-applicant. While working on the NIHR-Stroke Research Network (SRN) Rehabilitation Clinical Studies Group (CSG), he recognised that there was very little research into his experienced loss of confidence in life, a ‘hidden’ area of great importance to him. Through discussions with other stroke survivors it became apparent that this was a common experience. RC organised a small national seminar of researchers in the area of psychological rehabilitation and identity research, and brought CEH and FG together. This work is the result of that meeting. RC has been involved in national policy initiatives (eg, National Institute for Health Research (NIHR) and National Institute for Health and Care Excellence (NICE)) and two other service user members are representatives of the local Stroke Association and Different Strokes (two UK charities). We will recruit three additional service users from the Cambridge centre. One service user will be a member of the study steering group. All out of pocket expenses will be paid and time funded in accordance with INVOLVE’s recommendations,^58^ and working relationships will be informed by recommendations in INVOLVE’s briefing pack.^59^ Service users will be provided with regular email updates, be invited to provide comments via email and to attend three service user meetings over the course of the study. All service users will be invited to review drafts of the study report, contribute to writing the Plain English Summary and advise on dissemination. They will also be invited to comment on, contribute to, and lead on other outputs such as web blogs and articles in newsletters.

### Sample size considerations

A sample size of 64 will be adequate to estimate, to an acceptable degree of precision, the key parameters needed to address the study objectives. A questionnaire return rate of 80% would mean data at baseline and 1 month postintervention would be available for 51 participants. Estimating the recruitment rate will help us to plan the full-scale RCT. Precision of this estimate will be summarised using the distance from the estimate to the upper/lower limit of the 95% CI. With a total sample size of 64, the recruitment rate will be estimated with a precision of ±6% (assuming a recruitment rate of 30%). Assuming a questionnaire return rate of 80%, the return rate (in the short term) will be estimated with a precision of ±10%. We aim to estimate the SD of potential primary outcome measures in preparation for a formal sample size calculation for a larger RCT. Assuming these outcomes are standardised (SD of 1), the precision (as summarised by SE) will be 0.1 based on n=51 with follow-up data. For the full trial, the sample size calculation will be inflated appropriately to take into account potential clustering effects due to the group-based nature of the intervention.^60^ The aim is not to test the effectiveness of the intervention. However, we will take into account the magnitude of the estimated effect size (95% CI) when considering the plausibility of effect sizes to be used in the sample size calculation for the full trial.

### Participants

At each centre (Bournemouth and Cambridge), we aim to recruit 32 participants (64 in total). In each centre we will run two successive iterations of the HoS group intervention (in total: 4 blocks of 16 participants recruited, 2 blocks per centre). In each block of 16 participants recruited, half will be randomised to HoS plus usual care and the other half to usual care alone.

*Inclusion criteria*

- Diagnosis of stroke
- Aged 18 or above
- Physical, communication or cognitive symptoms from stroke at 5 days post-stroke
- Up to 2 years post-stroke
- Able to toilet independently (this could include people who use catheters/pads)—because the artist facilitators are not trained to support people to transfer
- Living in the community

*Exclusion criteria*

- Cognitive levels such that an individual would be unable to complete assessments even with support.
- Severe receptive aphasia which means that the person will not be able to comprehend the consenting process or engage with the HoS group
- Already receiving a psychiatric or clinical psychology intervention
- Living in a residential/nursing home

### Identification and recruitment

#### Bournemouth centre *(Royal Bournemouth and Christchurch Hospitals (RBCH) NHS Foundation Trust)*

Potential participants will be identified by stroke ward research staff at RBCH NHS Foundation Trust. They will either be sent or given an invitation letter, key facts sheet and reply slip by a member of the clinical team. They will be asked to return a reply slip in a prepaid envelope or email/telephone the Bournemouth centre research assistant if they are interested in finding out more about the study. If no reply from an individual is received, electronic records will be checked by the research nurse to ensure they have not died/become unwell before sending a reminder letter. If there is still no reply this will be noted in the site file. The research assistant will contact those who have expressed an interest in the study and answer any questions or queries they have. This will generally be via telephone (but will be face-to-face if the person has a communication disability). If they are still interested in taking part, they will be sent or given a set of Participant Information Sheets (PIS).

#### Cambridge Centre*(Cambridgeshire Community Services NHS Trust)*

In the Cambridge Centre, clinical staff from the stroke and community neurorehabilitation teams will identify potential participants in the community. Potential participants will be recruited using both the invitation letter approach as described above for the Bournemouth centre, and also a ‘consent to contact’ approach. For this, potential participants will be asked by a member of the clinical community team if they are interested in finding out more about the study, and if so will be asked to provide consent to be contacted by the Cambridge centre research assistant to obtain further information. A member of the clinical team will obtain this consent during a face-to-face consultation or verbally over the phone. If the individual remains interested, the research assistant will give them a set of PIS.

### Informed consent process

If an individual wishes to take part, the local research assistant will arrange to visit the person at home within 1 month prior to the start of the HoS group. Any unanswered questions relating to the study will be addressed and if the person remains eligible and still wishes to take part, they will be asked to complete and sign the consent form. All individuals are expected to give consent for themselves. Some individuals may have slight cognitive or communication difficulties due to their stroke. The research assistants at each centre will be Good Clinical Practice (GCP) trained and will have the skills to ensure that consent is fully informed.

### Randomisation

Randomisation will be achieved by means of a web-based system created by the Peninsula Clinical Trials Unit (PenCTU) in conjunction with the trial statistician. The website and database will be hosted by Plymouth University, and these are secured and backed up in accordance with their standard policies.

Participants will be allocated to intervention or control in a 1:1 ratio, using minimisation to balance the numbers of participants allocated to each group. Randomisation will be stratified by recruiting centre and stroke severity (Rivermead Motor Assessment—Gross motor subscale^61^ (≤6/≥7)). A stochastic element is included in the minimisation process to reduce the chances of predicting the randomisation outcome.

To perform randomisation the study research assistants will log on to the website using their unique username and password. Using the website they will be able to randomise participants individually or in batches. The intention is that batch randomisation will be performed when 12 or more participants have consented and undergone baseline assessment. Individual randomisation will then be performed to ‘top up’ the two trial arms if any further participants are recruited before the treatment groups start (randomising up to 18 in total).

### HeART of Stroke Group Intervention

Sessions will be held in community venues recommended by service users that have good accessibility, parking and public liability insurance.

Ten 2 h HoS group sessions will be held in a community venue over 14 weeks (table 1). Based on service user suggestions made when we were designing the intervention, session frequency will be tapered to support the postgroup transition (first 7 sessions held weekly, sessions 8–9 fortnightly and session 10 held 3 weeks later). It will be a closed group of 6–8 participants with a focus on the person rather than his/her stroke.

**Table 1:** HeART of stroke group intervention

Session number	Focus of session
1–3	Introductions and initial exploration
4–7	Group members encouraged to co-facilitate/engage in self-directed creative practice within sessions and at home
8–10	Ongoing sessions as well as opportunities for group community visits

The essential features of the group are the opportunity to be creative and the safe group atmosphere created and maintained by the artist facilitator. Within neuropsychological rehabilitation, it is hypothesised that establishing a safe place where clients feel understood and supported can facilitate self-development.^30^ ^62^ Creative activities allow access to internal resources and non-verbal/emotional as well as rational ways of knowing and build skills to foster self-esteem.^34^ ^63^ Members will be encouraged to (1) explore their sense of self and support others’ explorations; (2) be non-judgemental of self/others; (3) exercise personal choice; (4) develop a sense of play/improvisation.

Each week the artist facilitator will prepare resources in response to the group's creative interests and skills, including paints, drawing materials, clay, textiles and mixed-media. The group will be offered ‘stimulus’ pieces, such as books, poems, images, music and films, and be encouraged to bring pieces of interest to the group. They will be supported to explore their new lives through reflection, using imagination and engagement in arts-practice.^64^ Following each session, the artist facilitator will briefly document observations and reflect further to inform the selection of major provocation/stimulus materials for the next session.

### HoS artist facilitators

There will be one HoS artist facilitator per centre, each with more than 5 years’ experience in AfH work. They will be independent practitioners contracted to the NHS and with an honorary contract with their local Trust. At the start of the study they will receive training regarding study expectations and standardisation of the approach (CEH—Bournemouth; FG—Cambridge). For research purposes, they will have access to a professional with experience in stroke and psychology (CEH—Bournemouth, FG—Cambridge). Any support needed will be recorded to inform training/support needs for the future full trial. For research purposes only, the artist facilitators will Skype each other to ensure ongoing standardisation of the approach. This will not be needed in the future trial as artist facilitators will be able to use the guidebook produced.

### Skill set needed

- At least 5 years’ experience of facilitating creative Art for Health groups
- Ability to hold a safe space where individuals can express themselves in confidence
- Familiarity with mixed-media and arts in general
- Willingness to follow/support participants’ emerging interests
- Commitment to documenting/reflecting on sessions personally/within research group.

A researcher will be ‘on hand’ at the venue in case any additional help is needed (eg, participant unwell), but will not be directly involved in the session. The level of support provided will be assessed to determine the need for a second person being available in a future trial.

### Usual Care

In Bournemouth, support is provided by the Early Supported Discharge multidisciplinary team for 2–6 weeks after leaving hospital and then medical care via the GP, with a referral to the Stroke Coordinator. People with complex medical conditions are seen by Stroke Consultants as hospital outpatients. Ongoing rehabilitation needs are met by rehabilitation teams and in some areas day hospital service provision.

In Cambridgeshire, medical care is delivered via the GP and people with complex medical conditions are seen by Stroke Consultants as hospital outpatients. All can access support from the Stroke Association ‘Information, Advice and Support Coordinator’ and may receive additional therapy or support via one of three locality neurorehabilitation teams. Participants in both arms of the trial will receive usual care, and usual care will not be affected by involvement in the trial.

### Outcome measures and blinding

As one of the objectives of this feasibility study is to refine the selection of outcome measures for a subsequent full trial we have included a broad range of outcomes and are exploring the extent to which participants require support to complete them.

In the HoS arm, outcome measures will be administered within 1 month prior to the HoS group (baseline) and then approximately 1 month following its conclusion. For those allocated to the usual care arm, outcomes will be administered within an equivalent time frame. Outcome measures are all self-reported and presented in a booklet in a large font.

At baseline, outcome measures will be administered face-to-face by a research assistant in participants’ homes. At approximately 5 months postrandomisation (1 month post-HoS intervention) outcomes will be administered by post, or if needed, with face-to-face or telephone support from a blinded assessor. Support needs will be logged. Participants will be asked not to disclose their allocation arm to the blinded assessor. The assessor will log allocation beliefs and instances of unblinding.

#### Demographic/descriptor variables

Age, gender, marital status, educational qualifications, ethnicity, household composition, employment situation, comorbidities and medication will be recorded at baseline.

#### Stroke-related information

Type, side-of-stroke and time-since-stroke, mobility (Rivermead Motor Assessment—Gross Motor subscale^61^), upper limb impairment (Motricity Index^65^), communication ability,^66^ cognitive ability.^67^

##### Proposed Possible Primary Outcomes for a future trial

- Mental Well-being—Warwick and Edinburgh Mental Well-Being Scale (WEMWBS)^68^
- Anxiety and Depression—Hospital Anxiety and Depression Scale (HADS)^69^
- Quality of Life—ICEpop CAPability measure for Adults (ICECAP-A)^70^

##### Proposed Possible Secondary Outcomes for a future trial

- Self-esteem—Rosenberg Self-Esteem Scale (RSES)^71^
- Self-concept—Self-concept Head Injury Semantic Differential Scale (HISDIS)^72^
- Quality of Life—Medical Outcomes Short Form-36 (SF-36) (V.1)^73^

##### Process measures

- Views of recruiters
- Number of people approached, number eligible, number who agree to be contacted/to take part
- Short-term study retention rates (until 1-month postgroup time point)
- Group attendance rates/group sizes and composition, reasons for non-attendance (where known)
- Number of questionnaire reminders sent/participants requiring support to complete outcomes and levels/patterns of missing data
- Sense of belonging in the group at first, fifth and last HoS session (Doojse scale^74^)
- Number of participants in the HoS group who contact group members outside the group setting
- Postsession reflection notes kept by the HoS artist facilitators.

### Participant withdrawal from the intervention and/or research follow-up

Participants will be informed that they can withdraw from the study at any time without their care being affected in any way. If a participant withdraws consent to be included in research follow-up during the feasibility trial, the study research assistant at the centre will be informed and will contact the participant. If a participant in the HoS arm wishes to withdraw from the intervention, the research assistant will determine whether he/she also wishes to withdraw from the research follow-up. For those wishing to withdraw from the research follow-up, the research assistant will determine whether the participant is willing for data collected before withdrawal to be used at final analysis, or whether this information should be destroyed. No data will be used in the final analysis without a participant's consent.

### Data protection and data storage and management

All data will be handled, stored and protected in accordance with the Data Protection Act (1998) and the NHS Code of Confidentiality (2003). Data will be anonymised and only accessible to authorised staff working on the study.

Data processing, management, validation and quantitative analysis activities will be overseen by the Bournemouth University Clinical Research Unit and conducted in accordance with their standard operating procedures to ensure a clear audit trail and that relevant regulatory governance requirements are met.

The primary source of quantitative data will be questionnaire-based participant reported measures, but additional data will include recruitment logs kept by the Clinical Research Network funded researchers and by the study research assistants and attendance logs kept by the HoS artist facilitators. Data will be anonymised and single entered onto a password protected SPSS (V. 21) database stored on a secure backed-up university networked drive by the research assistant at the lead centre. A random sample of 20% of the data will be rekeyed (by ST) to check data quality. The resultant data will be cross-checked and differences resolved by referring back to the primary source. Any changes to data points will be documented to ensure a clear audit trail. Prior to the main analysis, statistical data checks will be carried out (eg, range checks). The database will then be closed to further changes.

Digital audio recordings of interviews and transcripts (see later) will be stored in secure password protected files on a backed-up university networked drive.

### Statistical analysis

This is a feasibility study and so the aim is not to provide a fully powered hypothesis test of the effectiveness of the intervention.^75^ Instead, analyses will focus on estimating key feasibility study parameters. We will also develop and test out data analysis procedures, with the particular aim of informing the statistical analysis plan for a full trial. Preliminary estimates of effect size (with 95% CIs) for potential primary outcome measures will be calculated so that these can inform the plausibility of the effect sizes used in future sample size calculations.

The main statistical analysis will be carried out using IBM SPSS software and will also include:

1. Producing a CONSORT diagram to represent numbers of stroke survivors eligible for inclusion, recruited, randomised and completed;
2. Descriptive statistics (with 95% CIs) will be used to summarise data relevant to recruitment, attrition, outcome measures, process measures, return rates and attendance at HoS sessions;
3. SDs (95% CI) of potential primary outcome measures will be estimated;
4. Rates and patterns of missing questionnaire data will be examined to inform a strategy for dealing with missing data in a full trial;
5. NHS and social care use will be summarised via descriptive statistics, and we will derive QALY estimates from SF-6D utility scores.

### Feasibility economic evaluation component

The future study will include a full within-trial economic evaluation, comparing the cost of delivering treatment in both arms in relation to quality adjusted life years (QALYs) in the primary analysis, and the primary clinical outcome of choice in secondary analysis. The primary analyses will take on the health and social care payer perspective, with secondary analysis from a societal perspective on costs.

In this feasibility economic evaluation, the data collection tools to collect resource use and costs for the future trial will be developed and refined. We will develop case report forms for the artist facilitators to complete with resources required to deliver the HoS intervention: preparation time, contact time and travel time, materials used, venue, numbers of participants attending the session and supervision required. Resources required to deliver care in both arms will be collected from a telephone-administered resource use questionnaire, administered to the participant or their carer, at 5 months postrandomisation, and a prospectively completed resource use log to use as a memory aid.^76^ Examples of resources that will be included are GP visits, informal care, productivity losses, use of social services and medications.

We will report a preliminary estimate of the cost of delivering the HoS intervention using a microcosting approach. We will use UK preference-based tariffs to the SF-6D and ICECAP-A generic scores to derive short-term quality adjusted life years (QALYs) estimates at follow-up per arm. Levels of missing data per cost category will also be reported. We will develop strategies to address missing data in poorly completed resource use categories for the future trial. Our experiences of data collection in the feasibility study will be useful in enabling refinement of the tools and methods to be used in the full-scale trial.

### Qualitative component

To gain valuable in-depth data about study processes and the HoS intervention,^77^ 12 (8 intervention; 4 control) face-to-face interviews (in participants’ homes) will be conducted across both centres by CEH at (1) postrandomisation but preintervention, and (2) after all the assessments are completed at 5 months postrandomisation (24 interviews in total). Purposive sampling will be used to capture variations that may influence perceptions, including age, gender, communication disability and severity of stroke.

Interviews will include: (1) *research aspects—*reasons for participation, acceptability of trial processes/outcome measures/feedback for improvement of participant experience/perceived willingness to complete longer term follow-ups (2) *intervention aspects* (intervention arm only)—expectations and experience of the intervention, acceptability of venue/intervention, ability/willingness to pay for own transport costs. (3) *stroke aspects* (both)—experience of well-being/self-confidence.

The artist facilitators will keep weekly postsession reflections. With participants’ permission, work created will be photographed by the artist facilitators to explore changes over time.

Interviews will be transcribed verbatim. Each transcript will be read and re-read in detail and codes related to the three aspects covered in the interview will be noted, namely; (1) research (2) intervention (3) stroke confidence/well-being. Each transcript will be read and coded independently by CEH, FR and KG. Following this, similarities and differences across transcripts will be noted and initial understandings will be made for each iteration. For simple aspects (such as the cost of transport), the analysis will be at the level of content analysis.^78^ For more complex aspects (such as participants’ expectations/experiences) themes will be produced and a thematic analysis carried out.^79^ During this process, the researchers will meet to discuss their codes, similarities and differences in order to highlight alternative interpretations which may have been missed. Finally, the similarities and differences across all four iterations will be discussed in relation to the aspects listed in (1), (2) and (3) as described above.

Each artist facilitator's weekly reflections will be discussed with the CI (CEH) and PI (FG), and key points shared with the research team to inform training and standardisation of the approach for the full trial.

### Project management and safety monitoring

The chief investigator (CI) will have overall responsibility for study conduct and will liaise closely with the Bournemouth University Clinical Research Unit. At the lead centre, CEH (CI), PT (study statistician) and ST (methodologist) will have regular meetings to discuss study progress. The research assistants at each centre will deal with the day-to-day management and coordination of the study, and will have regular meetings with the PI at each centre and will call on other research team members as appropriate. The CI, PI and research assistants will have Skype meetings every 2–3 weeks. A project management group comprising all coapplicants, study staff and PPI representatives will meet five times over the course of the study with service user group meetings held on three of those 5 occasions. The study steering group (independent representative, CI, PI, statistician, PPI representative and representatives of the sponsor) will meet bi-annually to monitor progress.

Adverse events (AEs) will be closely monitored and reported to a Core Safety Team (CEH, FG, PT and ST). During all telephone and face-to-face contact meetings, the study research assistants will ask participants if they have experienced any AEs and log these. The HoS artist facilitators will be asked to record any AEs and report these to the local study research assistant. The Core Safety team will consider the events and offer advice to the project team. The CI will assess an AE to establish if it is a serious adverse event (SAE) according to the National Research Ethics Service (NRES) definition. If the AE is not defined as serious it will be recorded on a case report form and stored in the site file, and the participant will be followed up by the research team as deemed appropriate and where required. Reports of related and unexpected SAEs will be submitted to the NHS Research Ethics Committee (REC) within 15 days of the CI becoming aware of the event using the ‘Report of SAE form for non-CTIMPs’ (V.3 April 2007), published on the NRES website. The sponsor will be notified within 24 h.

### Monitoring and audit

The study will be monitored and potentially audited by the Research and Development Departments of the Royal Bournemouth and Christchurch Hospitals NHS Foundation Trust and the Cambridgeshire Community Services NHS Trust.

## Ethics and dissemination

Ethics approval was granted by the Exeter NHS REC (Ref: 13/SW/0136) on 30 July 2013. Local Research and Development approval was granted by the Royal Bournemouth and Christchurch Hospitals NHS Foundation Trust (the Study Sponsor) on 6 May 2014 and by Cambridgeshire Community Services NHS Trust on 29 May 2014. Should any protocol changes be deemed necessary we will notify and seek advice from the study sponsor, and obtain appropriate ethical and regulatory reviews and permissions. We will use version control on the protocol and record any protocol amendments. The study will be conducted in accordance with the Research Governance Framework for Health and Social Care and Good Clinical Practice. Trust and university lone working policies will be followed. Artist facilitators will have access to clinical psychology support if required and we will record any support needs that arise.

It is not expected that participants attending the HoS group will become distressed during the intervention, but if this occurs the situation will be managed by the experienced HoS artist facilitators. There are few risks related to the HoS intervention. However, potential risks have been identified and detailed preventive measures and planned responses have been put in place.

The research team aims to publish at least two publications in peer-reviewed journals as well as articles in clinical news journals and service user led articles in relevant publications. Other dissemination activities will involve a presentation at the key national stroke conference (UK Stroke Forum) and to stroke survivors at the UK Stroke Assembly. Additionally, local dissemination events involving research participants, practitioners, service managers and stroke researchers will be held at each centre to discuss the findings and look ahead to the future trial.

The project will have an ongoing web presence at Bournemouth University, and we will use newsletters and social media to increase visibility and provide updates on progress.

## Discussion

In line with the Medical Research Council's (MRC) guidance,^80^ this study will evaluate the acceptability and feasibility of delivering a community HoS group intervention for people following stroke and of testing its clinical and cost-effectiveness in a future definitive RCT. Findings from this study will help determine whether it is feasible to recruit, randomise and retain participants. It will offer an opportunity to refine the intervention and produce delivery guidelines and a training package to support set-up and delivery in new centres, and will inform approaches for recruitment, assessment and analysis in preparation for the full-scale trial.
